# Fetal Skeletal Muscle Progenitors Have Regenerative Capacity after Intramuscular Engraftment in Dystrophin Deficient Mice

**DOI:** 10.1371/journal.pone.0063016

**Published:** 2013-05-09

**Authors:** Hiroshi Sakai, Takahiko Sato, Hidetoshi Sakurai, Takuya Yamamoto, Kazunori Hanaoka, Didier Montarras, Atsuko Sehara-Fujisawa

**Affiliations:** 1 Department of Growth Regulation, Institute for Frontier Medical Sciences, Kyoto University, Kyoto, Japan; 2 Department of Clinical Application, Center for iPS Cell Research and Application, Kyoto University, Kyoto, Japan; 3 Department of Reprogramming Science, Center for iPS Cell Research and Application, Kyoto University, Kyoto, Japan; 4 Laboratory of Molecular Embryology, Department of Bioscience, Kitasato University School of Science, Kanagawa, Japan; 5 Molecular Genetics of Development, Institut Pasteur, Paris, France; University of Minnesota Medical School, United States of America

## Abstract

Muscle satellite cells (SCs) are stem cells that reside in skeletal muscles and contribute to regeneration upon muscle injury. SCs arise from skeletal muscle progenitors expressing transcription factors Pax3 and/or Pax7 during embryogenesis in mice. However, it is unclear whether these fetal progenitors possess regenerative ability when transplanted in adult muscle. Here we address this question by investigating whether fetal skeletal muscle progenitors (FMPs) isolated from *Pax3^GFP/+^* embryos have the capacity to regenerate muscle after engraftment into Dystrophin-deficient mice, a model of Duchenne muscular dystrophy. The capacity of FMPs to engraft and enter the myogenic program in regenerating muscle was compared with that of SCs derived from adult *Pax3^GFP/+^* mice. Transplanted FMPs contributed to the reconstitution of damaged myofibers in Dystrophin-deficient mice. However, despite FMPs and SCs having similar myogenic ability in culture, the regenerative ability of FMPs was less than that of SCs in vivo. FMPs that had activated *MyoD* engrafted more efficiently to regenerate myofibers than MyoD-negative FMPs. Transcriptome and surface marker analyses of these cells suggest the importance of myogenic priming for the efficient myogenic engraftment. Our findings suggest the regenerative capability of FMPs in the context of muscle repair and cell therapy for degenerative muscle disease.

## Introduction

The muscular dystrophies are a group of inherited skeletal muscle disorders that are characterized clinically as progressive skeletal muscle weakness and wasting [1]. The most common and severe form of muscular dystrophy is Duchenne muscular dystrophy (DMD) [2], caused by the mutation or deletion of the *Dmd* gene that encodes the structural protein dystrophin [2], [3]. Although several new approaches are being developed to retard the progression of symptoms of DMD, there is as yet no cure [4], [5]. Cell transplantation therapy is considered a promising approach to replace the abnormal skeletal muscle tissue of individuals with DMD with donor cells that express the missing dystrophin protein [5], [6].

In particular, the therapeutic myogenic potential of satellite cells (SCs) is noteworthy. Skeletal muscle SCs are mononuclear cells that reside in their niche, underneath the basal lamina of multinucleated myofibers [7]. They are mitotically quiescent cells that begin to proliferate upon myofiber injury or during exercise to expand a population of skeletal muscle progenitors required to reconstruct new myofibers [8]–[11]. SCs without a *Dmd* mutation engraft into the muscle of DMD model mice and contribute to the regeneration of dystrophin positive myofibres [12]–[14]. Although these studies suggest the regenerative ability of SCs for DMD, the mechanisms that control the regenerative ability of SCs when engrafted into muscle tissues have not been defined.

During embryonic skeletal muscle development, cells expressing both the paired/homeodomain genes *Pax3* and *Pax7* in the dermomyotome delaminate into the myotome and begin to express myogenic regulatory factors such as MyoD or Myogenin, leading to the formation of skeletal muscle [15]–[16]. Pax3 positive cells in the hypaxial somite migrate into developing limbs and begin to express the myogenic regulatory factors to found the skeletal muscle masses of the limb [15], [17], [18]. Undifferentiated, mononucleated progenitors expressing Pax3 or Pax7 are first found located between the basal lamina and plasma membrane of skeletal muscle fibers at fetal stage [19]–[20]. Pax7, and Pax3 in a subset of muscles, marks quiescent SCs in adult muscle [21]. In spite of detailed knowledge about the origin of SCs during development, the regenerative myogenic ability of these skeletal muscle progenitors for the rescue of DMD skeletal muscle fibers has not been explored.

In this study, we show that Pax3 positive cells isolated from wild-type fetal muscle, named fetal skeletal muscle progenitors (FMPs), have the capacity to regenerate dystrophin positive myofibres after engraftment into regenerating muscle of DMD-model mice. However, FMPs have a diminished capacity to regenerate muscle in vivo, compared to SCs, despite having an equal capacity to enter the myogenic program in vitro. To define the molecular mechanisms required for the acquisition of regenerative capacity by FMPs, we further evaluated the effect of *MyoD* expression in FMPs by genetic approaches. We find that *MyoD* expression enhances the regenerative capacity of FMPs cell-autonomously. These results, together with transcriptions and cell surface marker analyses, suggest the involvement of primed myogenesis in the efficient contribution of FMPs in regenerative myogenesis after engraftment.

## Materials and Methods

### Ethics Statements

All animal experiments were carried out according to the Regulations of Animal Experimentation at Kyoto University. The protocol was approved by the Animal Research Committee of Kyoto University (Permit Number: J-6). All injections were performed under anesthesia, and all efforts were made to minimize suffering. Mice were humanely sacrificed prior to tissue collection.

### Mice

The following mouse lines were used to obtain myogenic cells: *Pax3^GFP/+^* [20], *Rosa26^CAG−LSL−tdTomato/+^* (Jackson Laboratory, Bar Harbor, ME; stock number 007914, *R26R^RFP^*). The BAC *MyoD-Cre-IRES-nlacZ* transgenic line (*MyoD-Cre*) is described in Fig. S1. A BAC containing *MyoD* genomic DNA (−100 kb/+100 kb: clone RP23-46A24 purchased from BACPAC resource center, CHORI) was used for targeting with a *Cre-IRES-nlacZ* reporter into the ATG site of *MyoD*. All BAC recombineering was performed with SW105 *Escherichia coli* strains [22]. The *DMD-null* line [23], which completely lacks dystrophin without any sporadic revertant dystrophin-positive fibers, was used as host mice. To avoid immune rejection of donor cells allowing long-term engraftment, the KSN/Slc line (*Foxn1^−/−^*, or *nude*, purchased from Japan SLC, Hamamatsu, Japan) were crossed with the *DMD-null* line to obtain *DMD-null;nude* compound mice.

### Preparation of Cells for Flow Cytometry Analysis and Cell Sorting

Fetal skeletal muscle progenitors (FMPs) were obtained from the limbs and diaphragm of E16.5 fetuses of *Pax3^GFP/+^ or Pax3^GFP/+^;MyoD-Cre;R26R^RFP^* compound mice. To isolate FMPs from the limbs, the skin was carefully removed to avoid contamination with (Pax3)GFP-positive (+) cells present in the skin [24]. Tissues were dissociated with 0.08% collagenase (Sigma, St Louis, MO) and 0.08% trypsin (Roche, Basel, Switzerland) in DMEM/F12 supplemented with GlutaMAX (Gibco, Carlsbad, CA) at 37°C for 40 minutes. Dissociated cells were resuspended with 1% fetal bovine serum (FBS; Gibco) in DMEM-high glucose (Gibco) and filtered with 35-µm cell strainers (BD, Franklin Lakes, NJ). Satellite cells (SCs) were prepared from abdominal muscles and diaphragms of 8–12 week old *Pax3^GFP/+^* mice by enzymatic dissociation as previously described [14]. For live cell sorting, single-cells were stained with 1 µg/ml propidium iodide (PI) to exclude PI+ dead cells. Cell sorting was performed with FACSAria II Cell Sorter (BD). The complete (Pax3)GFP+ fraction was taken for analysis using FlowJo (Tree Star, Ashland, OR).

### Characterization of FMPs and SCs in vitro

Isolated FMPs and (Pax3)GFP+ SCs were resuspended in the growth medium DMEM/F12 (Gibco) containing 20% FBS (Gibco) and 2% Ultroser G (Pall, Port Washington, NY). Cells were cultured in 35-mm dishes coated with 2% gelatin at 5×10^3^ cells per dish. Four days later, the medium was changed to the differentiation medium, which consisted of DMEM/F12 with 2% horse serum (Sigma).

For immunocytochemical analysis, cultured cells were fixed with 4% paraformaldehyde (PFA) and permeabilized with 0.2% Triton X100, 50 mM NH_4_Cl in PBS. Cells were incubated with 5% Blocking One (nacalai tesque, Kyoto, Japan). The following antibodies were used as primary antibodies: anti-MyoD (1∶100, Santa Cruz Biotechnology, Santa Cruz, CA), anti-Myogenin (1∶100, DAKO, Glostrup, Denmark), anti-Troponin T (1∶250, Sigma). Secondary antibodies were coupled to flurochromes Alexa 568 or 647 (1∶300, Molecular Probes, Carlsbad, CA). 4,6-Diamidino-2-phenylindole (DAPI, 1∶10,000, Molecular Probes) was used to counter-stain nuclei. For quantification, at least 500 cells in culture were counted from randomly chosen fields for each stage.

### Engraftment of Isolated FMPs and SCs into Injured Muscle Tissues

*DMD-null* or *DMD-null;nude* host mice were injected with 50 µl of 10 µM cardiotoxin (Sigma) the day before transplantation. For engraftments, purified FMPs and SCs (2×10^4^ cells per 20 µl of PBS) were injected into tibialis anterior (TA) muscles of anesthetized host mice. Control TA muscles were injected with medium as a negative control.

TA muscles were removed two weeks after transplantation. Injected muscles were frozen in liquid nitrogen-chilled isopentane. For immunofluorescence staining, serial 10 µm cryosections were collected and blocked with 5% Blocking One in PBS. Antibody for dystrophin (1∶500, Abcam, Cambridge, UK) was diluted in 5% Blocking One in PBS. Alexa fluor 568 goat-anti-rabbit IgG was used as a secondary antibody. DAPI (1∶10,000, Molecular Probes) was used to counter-stain nuclei. Stained tissues were photographed with a Zeiss Axio Imager Z1 (Carl Zeiss, Oberkochen, Germany). For quantification of dystrophin positive fibers, serial transverse sections were cut throughout the entire TA muscle. Each TA muscle generated 20–25 slides, each slide consisting of 20–25 serial sections. Five different slides were stained for dystrophin. The one section in the slide with the maximum number of dystrophin positive myofibers for each animal was counted. Graphs display the mean ± s.e.m. for each engrafted animal.

### Immunohistochemistry and Quantification of Engrafted FMPs and SCs

To maintain GFP fluorescence in the tissue, TA muscles were fixed in 1% paraformaldehyde, 0.1% Triton X-100 in PBS at 4°C for 2 hours and incubated in 15% sucrose in PBS at 4°C overnight. The TA muscles were embedded in Frozen Section Compound (Leica Microsystems, Wetzlar, Germany) and were frozen in liquid nitrogen. For immunofluorescence staining, serial 10 µm cryosections were collected and treated with 10% goat serum in PBS. Antibodies for GFP (1∶200, Molecular Probes), dystrophin (1∶500, Abcam), and laminin (1∶500, Enzo Life Sciences, Farmingdale, NY) were diluted in 0.5% Triton X-100 in PBS and were incubated overnight at 4°C. Alexa fluor 488 goat-anti-chick, 568 goat-anti-rabbit, and 647 goat-anti-rat (1∶500, Molecular Probes) IgGs were used as secondary antibodies. Stained tissues were photographed with a Leica TCS-SP5 Confocal Microscopy (Leica Microsystems). For quantification of (Pax3)GFP+ cells underneath laminin and outside the muscle fiber, the entire TA muscle was sectioned. Each TA muscle generated 20 slides, each slide was about 200 µm away from the previous slide. Each slide consisted of nine serial sections, and four different slides were stained for GFP, dystrophin, and laminin. The average number of (Pax3)GFP+ cells were counted in nine sections obtained from four different slides of TA from *n* = 5 mice.

### Immunohistochemistry of Fetal Limbs Sections

Limbs from fetuses collected at E16.5 days post coitus were fixed with 4% PFA at room temperature for 2 hours followed by overnight immersion in 20% sucrose in PBS at 4°C. Fixed tissue was embedded in Optimal Cutting Temperature (O.C.T.) Compound (Sakura Finetek Japan, Tokyo, Japan) for cryosections. Immunohistochemistry was carried out using the following antibodies: anti-GFP (1∶500, Millipore, Billerica, MA) and anti-MyoD (1∶200, Santa Cruz Biotechnology). Alexa fluor 488 goat-anti-rabbit, goat-anti-chick, 568 goat-anti-rabbit, and 647 goat-anti-rat (1∶500, Molecular Probes) IgGs were used as secondary antibodies. Stained tissues were photographed with a Leica TCS-SP5 Confocal Microscopy (Leica Microsystems). For quantitative analyses of immunostained tissue, at least 100 (Pax3)GFP+ cells from randomly chosen fields in the limbs were counted from three fetuses.

### Immunocytochemistry of Freshly Isolated FMPs and SCs

FMPs and (Pax3)GFP+ SCs were collected by cell sorting and plated on gelatin-coated dishes for 30 minutes to allow attachment. For immunocytochemistry, cells were fixed with 4% PFA and processed for immunostaining as above. For quantitative analyses of immunostained cells, at least 100 (Pax3)GFP+ cells in culture from randomly chosen fields were counted from three independent experiments.

### Transplantation of Sorted MyoD-positive and -negative FMPs

To maintain RFP fluorescence in the tissue, transplanted TA muscles were fixed with 2% PFA at 4°C for 1 hours followed by washing with PBS at room temperature for 30 minutes. Fixed TA was frozen and processed for immunostaining with anti-dystrophin antibody as mentioned above. Quantitative analyses of dystrophin positive fibers were carried out as mentioned above.

### Profiles of Cell Surface Markers in FMPs and SCs

Cells were washed in 500 µl of Hanks' Balanced Salt Solutions (HBSS) with 1% bovine serum albumin (BSA). We used the following primary antibodies on 10^6^ cells in 100 µl of HBSS 1% BSA: biotinylated anti-CD34 (1∶100, clone RAM34, eBioscience, San Diego, USA), phycoerythrin (PE)-conjugated anti-CD184 (1∶40, clone 2B11/CXCR4, eBioscience), PE-conjugated anti-c-Met (1∶80, clone eBioclone 7, eBioscience), PE-conjugated anti-Sca-1 (1∶400, clone D7, eBioscience). For secondary staining, streptavidin coupled to APC, APC-conjugated goat anti-mouse IgG were used respectively. Flow cytometry analysis was preformed with a FACSAria II Cell Sorter.

### Quantitative Real-time Reverse Transcription Polymerase Chain Reaction Analysis

Total RNA was prepared using the RNeasy Plus Micro kit (Qiagen, Hilden, Germany) from freshly isolated cells. Synthesized cDNA was prepared from total RNA samples using SuperScript III kit (Invitrogen) with random hexamers for quantitative real-time reverse transcription polymerase chain reaction (qRT-PCR). All qRT-PCR reactions were carried out in triplicate using Power SYBR Green PCR Master Mix (Applied Biosystems, Carlsbad, USA) and StepOne Real Time PCR System (Applied Biosystems). All qRT-PCR results were normalized to the expression level of ribosomal protein L13A (Rpl13A) as a control gene. Primer sequences (5′ to 3′) are listed in the Table S1.

### Statistics

Statistical analysis was performed with R software using Welch Two Sample t-test, 2-sample test for equality of proportions, and Bonferroni test.

## Results

### FMPs are more Heterogeneous than SCs

**Figure 1:**
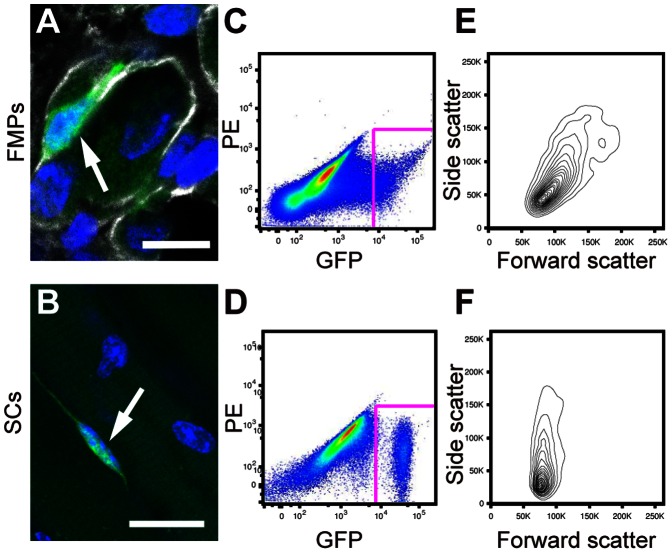
FMPs are more heterogeneous than SCs. (**A**) Immunohistochemistry of a FMP on longitudinal sections of limbs at E16.5 for GFP (green), Laminin (white). Nuclei were stained with DAPI. An arrow indicates a GFP-positive FMP. Scale bar = 10 µm. (**B**) Adult myofibers (nuclei were stained with DAPI) isolated from the diaphragms of the *Pax3^GFP/+^* line. An arrow indicates a GFP-positive SC. Scale bar = 25 µm. (**C,D**) Representative fluorescence-activated cell sorting profiles for (Pax3)GFP+ cells from fetuses (C) and adult muscle (D). (**E,F**) Forward scatter and side scatter profiles of (Pax3)GFP cells gated in (C) and (D). FMPs, fetal skeletal muscle progenitors; SCs, satellite cells.

To obtain fetal skeletal muscle progenitors (FMPs), (Pax3)GFP-positive (+) cells were isolated by fluorescence activated cell sorting (FACS) from limbs and diaphragm of E16.5 fetuses from *Pax3^GFP/+^* mice (Fig. 1A). Satellite cells (SCs) expressing Pax3 were prepared from abdominal muscle and diaphragm of adult *Pax3^GFP/+^* mice as previously reported (Fig. 1B) [14]. (Pax3)GFP+ cells represented 1–6% of the total mononuclear cells isolated from the tissue of fetal limbs and adult skeletal muscle fibers (Fig. 1C,D). Isolated SCs were homogenous, with low-variation in forward scatter (FSC) and side scatter (SSC) gating, indicating small and non-granular cells, as previously reported (Fig. 1F) [14]. In addition, SCs homogenously expressed GFP at high levels (Fig. 1D). In contrast, FMPs were heterogeneous with respect to the intensity of GFP expression (Fig. 1C), as well as FSC and SSC profiles (Fig. 1E). Taken together these findings indicate that FMPs are more heterogeneous than SCs.

### FMPs have Similar Myogenic Potential to SCs in vitro

**Figure 2:**
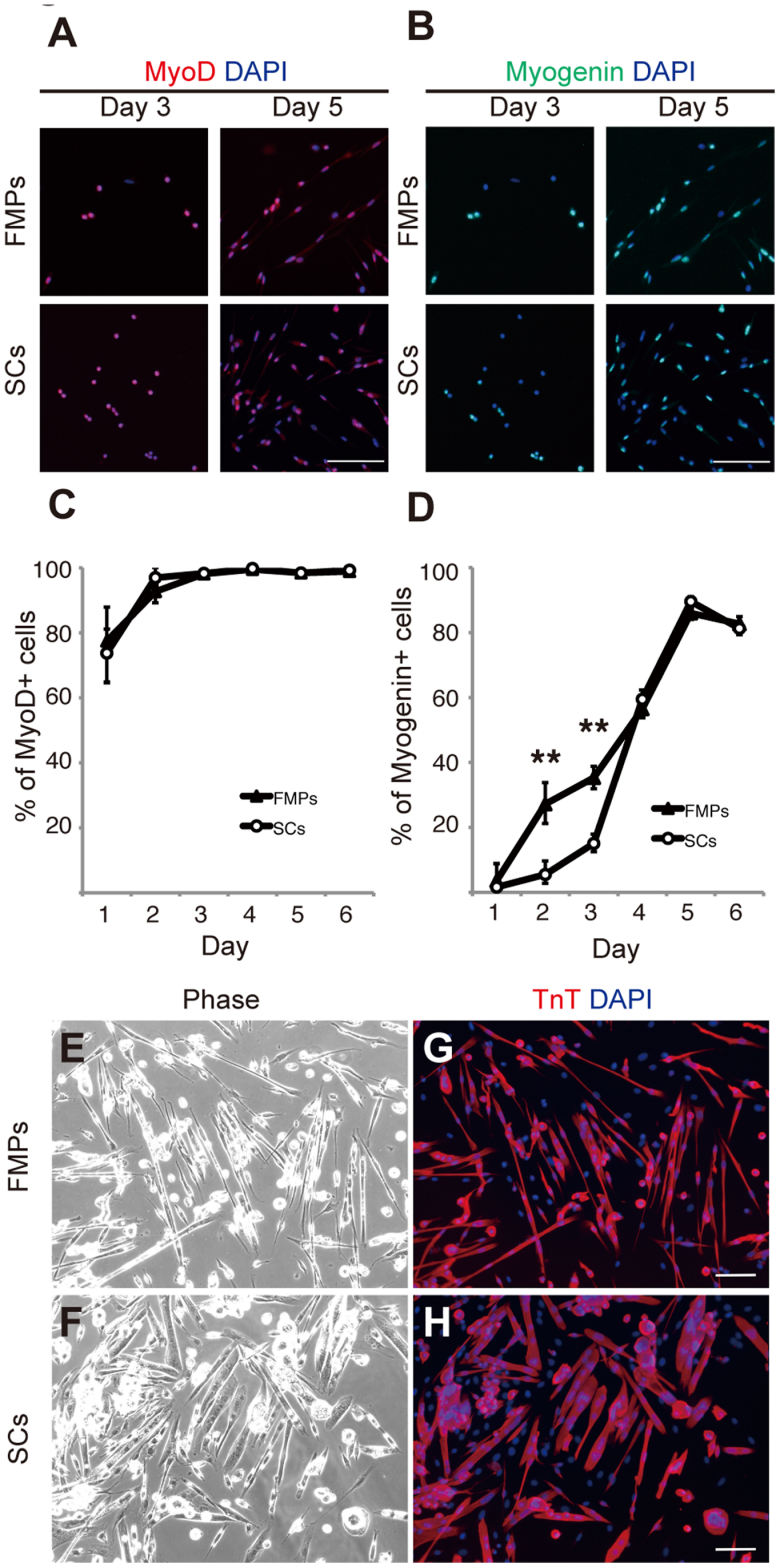
FMPs and SCs show similar myogenic differentiation potential in vitro. (**A,B**) Immunocytochemistry of FMPs and SCs for MyoD (A) and Myogenin (B) during culture (days 3 and 5). DAPI was used for to stain nuclei. Scale bar = 50 µm. (**C,D**) Quantitative analysis of MyoD (C) and Myogenin (D) in cultured FMPs and SCs. Data are reported as mean and s.d. of 500–1000 cells per staining from three independent experiments. P-values indicated on figures are <0.01 (**). (**E,F**) Phase contrast micrographs of FMPs (E) and SCs (F) after 7 days in culture. (**G,H**) Immunocytochemistry of FMPs (G) and SCs (H) shown in (E,F) for Troponin T and DAPI. Scale bar = 50 µm. FMPs, fetal skeletal muscle progenitors; SCs, satellite cells.

We next assessed the myogenic differentiation potential of FMPs in vitro and compared them with those of SCs. Sorted FMPs and SCs were plated on gelatin-coated dishes and stained with MyoD and Myogenin after 1 to 6 days in culture (Fig. 2A–D). FMPs and SCs had a similar temporal expression pattern of MyoD in culture (Fig. 2A,C). In terms of Myogenin expression, 5–15% of SCs were positive for Myogenin on day 2 and 3, respectively compared with 27–35% of FMPs (Fig. 2B,D). After 4–6 days in culture, however, Myogenin expression in FMPs was comparable to that of SCs (Fig. 2B,D). After 7 days in culture, FMPs and SCs generated multinucleated myotubes (Fig. 2E,F) expressing Troponin T (Fig. 2G,H). Therefore, FMPs and SCs were highly myogenic in terms of MyoD expression and differentiation into myotubes while part of the FMP population differentiated more rapidly than SCs.

### FMPs Engraft *DMD-null* Skeletal Muscles in vivo

**Figure 3:**
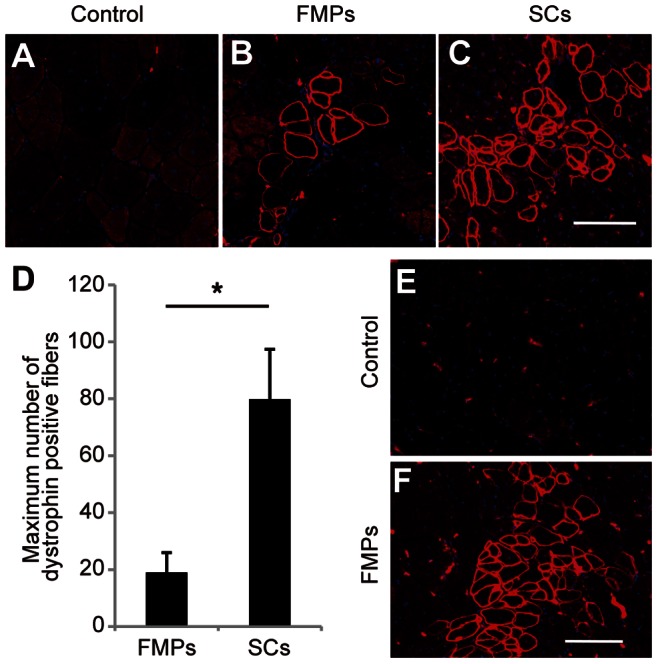
Engrafted FMPs restore dystrophin expression in *DMD-null* mice. (**A–C**) Immunostaining for dystrophin in TA muscles of *DMD-null* mice injected with medium (A), FMPs (B), and SCs (C) 2 weeks after intramuscular engraftment. Scale bar = 100 µm. (**D**) Quantification of dystrophin+ fibers in TA engrafted with FMPs (*n* = 7 recipient mice) and SCs (*n* = 7 recipient mice). Data is reported as the mean and s.e.m. from all engrafted mice. P-value indicated on the figure is <0.05 (*). (**E,F**) Immunostaining for dystrophin in TA muscles of *DMD-null;nude* mice injected with medium (E) and FMPs (F), 24 weeks after engraftment. Scale bar = 100 µm. FMPs, fetal skeletal muscle progenitors; SCs, satellite cells; TA, tibialis anterior.

To compare regenerative capacities, FMPs and SCs (2×10^4^ cells) were injected into cardiotoxin-injured tibialis anterior (TA) muscles of dystrophin knock-out mice (*DMD-null* mice) [23]. The expression of dystrophin in host muscle fibers was evaluated two weeks after engraftment. Immunostaining showed the presence of dystrophin-positive (+) fibers in host muscle sections from *DMD-null* mice engrafted with SCs and FMPs (Fig. 3B,C). No dystrophin+ fibers were present in non-grafted TA muscles (Fig. 3A). Serial cryosections of the TA after FMP transplantation showed continuous dystrophin expression (Fig. S2). Quantification of dystrophin+ fibers in transplanted *DMD-null* mice showed that SCs had higher efficiency of engraftment than FMPs (79.8±17.5 fibers with SCs, *n* = 7 independent experiments, and 19±6.9 fibers with FMPs, *n* = 7 independent experiments, Fig. 3D). To determine whether the engraftment of FMPs was sustained, we investigated the restoration of dystrophin expression in skeletal muscle fibers of host mice at 24 weeks after transplantation into *DMD-null;nude* mice [25]. Dystrophin+ fibers were also detected in muscles of *DMD-null;nude* mice (*n* = 3 independent experiments) 6 months after transplantation (Fig. 3F), whereas the control mice presented no dystrophin+ myofibers (Fig. 3E). No tumor formation was observed in mice up to 6 months after the engraftment (data not shown). These observations suggest that FMPs were able to contribute to regenerated fibers over a long period but that engraftment of skeletal muscles was less efficient than SCs.

### Transplanted FMPs Contribute to the SC Compartment

**Figure 4:**
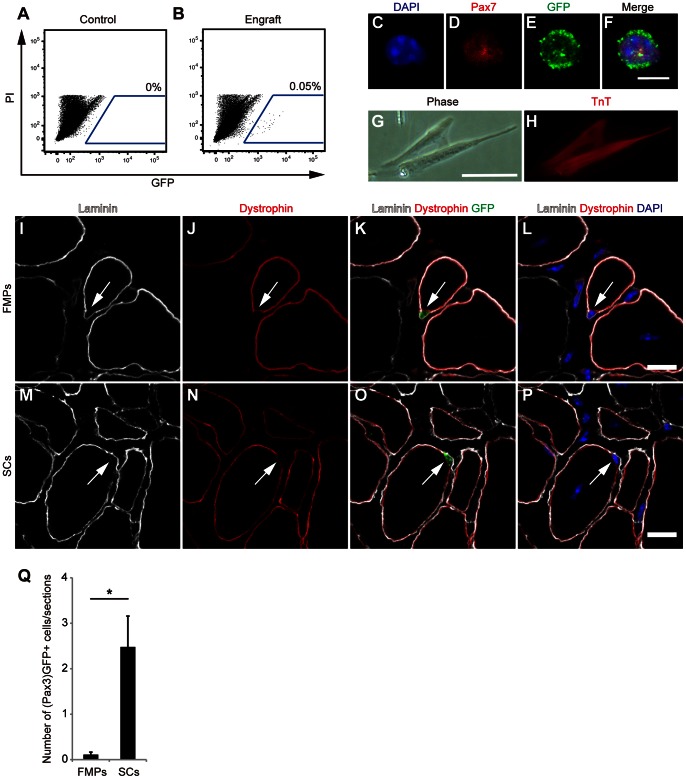
Recovery of (Pax3)GFP+ cells from FMP- and SC-engrafted TA muscles. (**A,B**) A representative fluorescence-activated cell sorting profile for (Pax3)GFP+ cells from control (A) and FMP-engrafted (B) TA muscle. The percentage of cells that express GFP is indicated. (**C–F**) Immunocytochemistry for Pax7 (D) and GFP (E) of freshly isolated (Pax3)GFP+ cells from FMP-engrafted TA muscles. Scale bars = 5 µm. (**G,H**) Phase contrast micrographs (G) and immunocytochemistry with anti-Troponin T antibodies (H) of isolated (Pax3)GFP+ cells cultured in proliferation and differentiation conditions for 6 days. Scale bar = 50 µm. (**I–P**) Immunostaining for laminin, dystrophin, and GFP in TA muscles of *DMD-null* mice injected with FMPs (I–L) and SCs (M–P) 2 weeks after intramuscular engraftment. Arrows indicate (Pax3)GFP+ cells underneath laminin and outside of dystrophin. Scale bar = 10 µm. (**Q**) Quantification of (Pax3)GFP+ cells underneath laminin and outside of dystrophin in TA engrafted with FMPs (*n* = 5 recipient mice) and SCs (*n* = 5 recipient mice). Data is reported as the mean and s.e.m. from all engrafted mice. P-value indicated on the figure is <0.05 (*). FMPs, fetal skeletal muscle progenitors; SCs, satellite cells; TA, tibialis anterior.

(Pax3)GFP+ SCs isolated from the diaphragm retain their (Pax3)GFP+ identity in the environment of engrafted TA muscle, where endogenous SCs rarely express Pax3 [14]. To examine whether FMPs could also retain their (Pax3)GFP-expression in the engrafted muscle, the host TA muscles were analyzed by flow cytometry two weeks after engraftment. Although no (Pax3)GFP+ cell was detected in control TA muscles (Fig. 4A), (Pax3)GFP+ donor cells could be detected from the engrafted TA muscles (Fig. 4B). Freshly sorted donor-derived (Pax3)GFP+ cells were positive for Pax7 (Fig. 4C–F). In addition, these cells also expressed TroponinT after 6 days in culture (Fig. 4H).

To compare the properties of FMPs and SCs to occupy the satellite cell-niche of the engrafted muscle, we performed immunostaining of (Pax3)GFP, laminin, and dystrophin in FMP- or SC-transplanted TA muscles of *DMD-null* mice two weeks after transplantation. Engrafted (Pax3)GFP+ cells were located under the basement membrane (inside laminin) and outside of the muscle fiber (outside of dystrophin), in both FMP- and SC-transplanted muscles (Fig. 4I–P). A higher number of (Pax3)GFP+ cells occupying the satellite cell-niche was found in SC-transplanted TAs than FMP-transplanted ones (2.4±0.6 GFP+ cells/section with SCs, *n* = 5 recipient mice, and 0.1±0.05 GFP+ cells/section with FMPs, *n* = 5 recipient mice, Fig. 4Q). These results show that transplanted FMPs contribute to the adult muscle SC compartment though less efficiently than SCs.

We also isolated (Pax3)GFP+ cells from embryonic muscle (Fig. S3A–C). These cells did not survive in culture (data not show) or did not show significant levels of engraftment (Fig. S3D,E), in keeping with a previous report [26]. We therefore conclude that fetal but not embryonic muscle progenitors have the potential to repair adult skeletal muscle.

### MyoD Protein is Expressed in FMPs, but not in SCs

**Figure 5:**
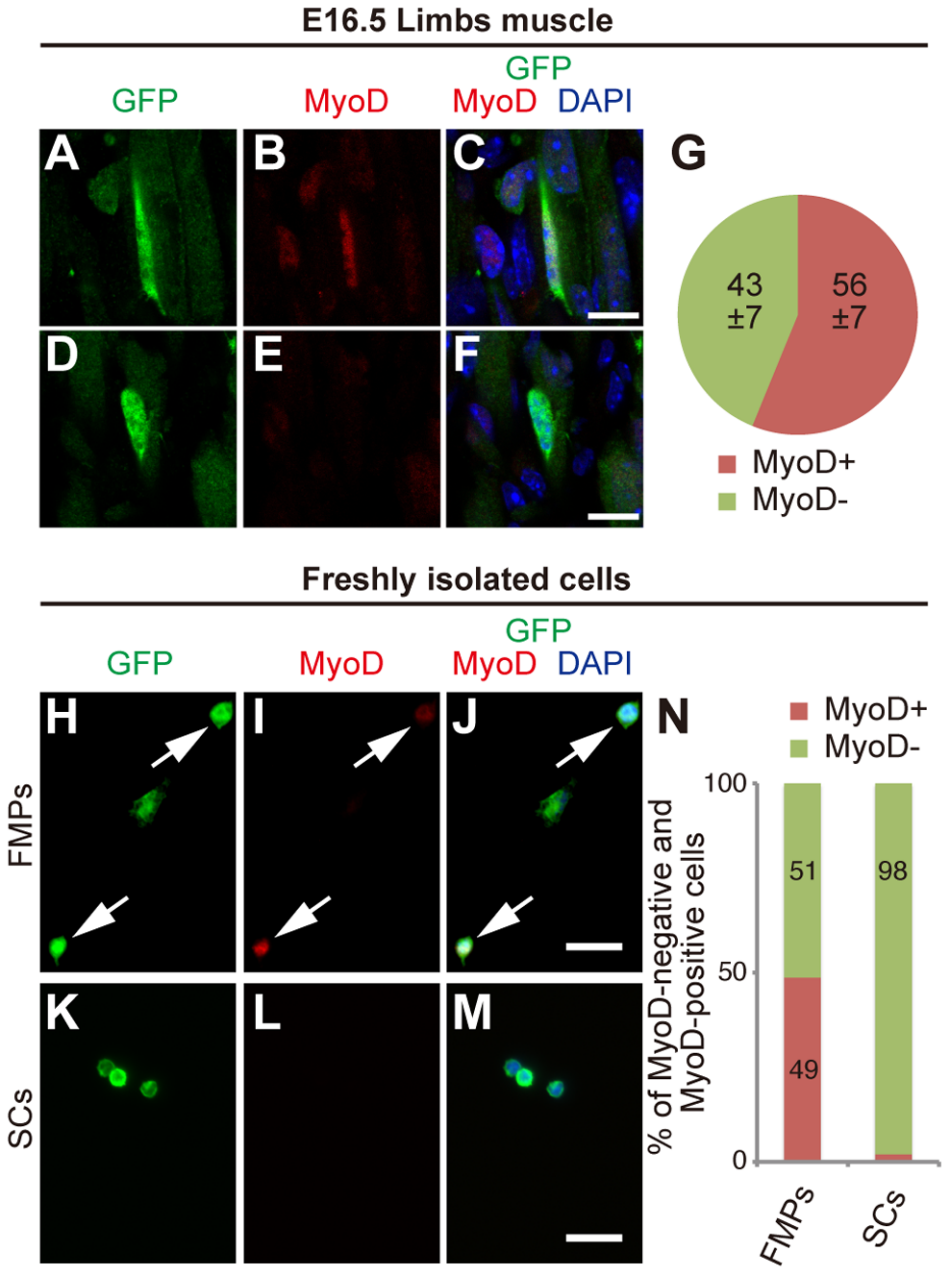
FMPs express MyoD in developing limbs. (**A–F**) Immunohistochemistry of FMP cells on longitudinal sections of limbs at E16.5 for GFP (A,D), MyoD (B,E) and merged with DAPI (C,F). MyoD-positive (A–C) and -negative (D–F) FMPs are shown. Scale bar = 10 µm. (**G**) Percentage of MyoD-positive and MyoD-negative cells in (Pax3)GFP+ cells in limbs at E16.5 with data reported as the mean and s.d. of three fetuses. (**H–M**) Immunocytochemistry of freshly isolated FMPs (H–J) and SCs (K–M) for GFP (H,K), MyoD (I,L), and merged with DAPI (J,M). Arrows in H–J indicate MyoD-positive FMPs. Scale bar = 20 µm. (**N**) Percentage of MyoD-positive and MyoD-negative cells of freshly isolated FMPs and SCs (*n = *100 cells par condition). FMPs, fetal skeletal muscle progenitors; SCs, satellite cells.

We next examined for factors that account for the different engraftment efficiencies between FMPs and SCs. Expansion of SCs in culture before engraftment results in the activation of MyoD protein expression and reduced regenerative capacity [14], [27]. Therefore, the lower regenerative ability exhibited by FMPs, compared to freshly isolated SCs, could be due to higher expression of MyoD protein. We examined the ratio of MyoD protein-positive cells in FMPs of the limbs, of fetuses at E16.5 of *Pax3^GFP/+^* mice. Half (56±7%) of (Pax3)GFP+ cells detected in limb muscles expressed MyoD (Fig. 5A–E,G). Next, we investigated whether these findings in limb muscles were also observed in the freshly isolated cells from adult and fetal muscles after cell-sorting. Most freshly isolated SCs expressing (Pax3)GFP were negative for MyoD (Fig. 5K–L for immunostaining and Fig. 5N for quantification analysis), in agreement with other reports [27], [28]. In contrast, half of isolated FMPs expressed MyoD (Fig. 5H–J,N). This reflects the developmental program of skeletal muscle progenitors entering the limbs, which are marked by (Pax3)GFP, and about half of these progenitors have already entered the myogenic program, marked by MyoD expression, and will contribute to the formation of new muscle fibers [20].

### MyoD-positive FMPs have more Regenerative Ability than MyoD-negative FMPs

**Figure 6:**
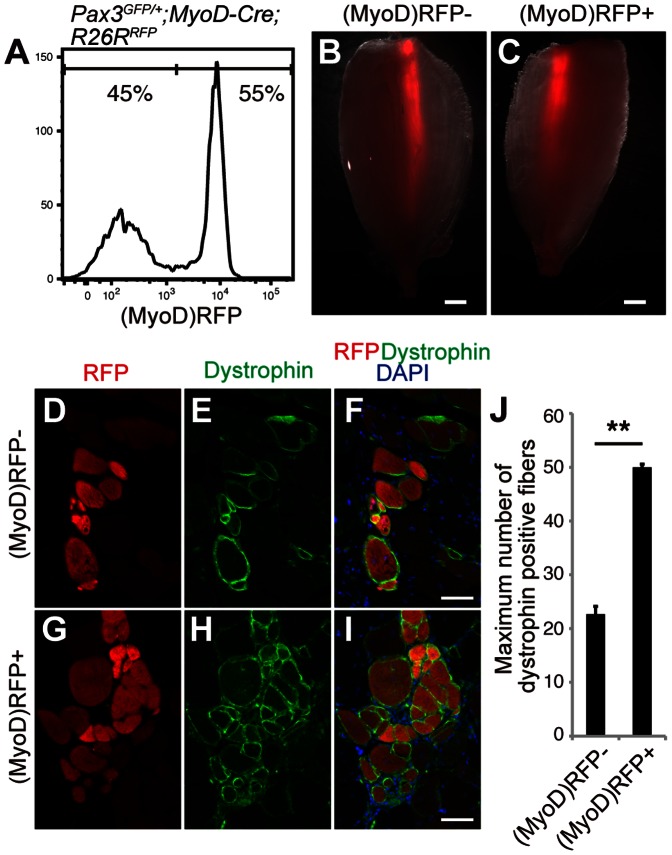
MyoD-positive FMPs have more regenerative ability than MyoD-negative FMPs. (**A**) Representative fluorescence-activated cell sorting profiles for (MyoD)RFP- and (MyoD)RFP+ cells from *Pax3^GFP/+^;MyoD-Cre;R26R^RFP^* fetus. (**B,C**) A fluorescence stereomicroscope view of RFP fluorescence in TA muscles of *DMD-null* mice transplanted with (MyoD)RFP- (B) and (MyoD)RFP+ FMPs (C). Scale bar = 2.5 mm. (**D–I**) Immunohistochemistry for RFP (D,E), dystrophin (E,F), and merged with DAPI (F,I) in (MyoD)RFP- (D–F) and (MyoD)RFP+ (G–I) FMP engrafted TA muscles. Scale bar = 20 µm. (**J**) Quantification of dystrophin+ fibers in TA muscles engrafted with (MyoD)RFP- and (MyoD)RFP+ FMPs. Error bars represent the mean and s.e.m. from 3 engrafted mice. P-value indicated on the figure is <0.01 (**). FMPs, fetal skeletal muscle progenitors; TA, tibialis anterior.

We then examined whether MyoD expression in FMPs also affected their efficiency of engraftment. We isolated FMPs from *Pax3^GFP/+^;MyoD-Cre;R26R^RFP^* mice, in which all descendants that had expressed *MyoD-Cre* at any time point before analyses were marked by the expression of RFP (Fig. S1). We obtained (Pax3)GFP+ fractions from fetuses of *Pax3^GFP/+^;MyoD-Cre;R26R^RFP^* mice at comparable proportions to those from *Pax3^GFP/+^* mice (Fig. 6A and Fig. S4). Half of the (Pax3)GFP+ cells were positive for RFP, which was comparable to immunostaining for MyoD of FMPs in vivo and in vitro (Figs 5, 6A). Most of freshly isolated (MyoD)RFP+ FMPs were positive for MyoD protein (Fig. S4). This reflects that most (MyoD)RFP+ cells at fetal stage express MyoD whereas (MyoD)RFP+ SCs in adult muscles are negative for MyoD (data not shown and [29]). The (MyoD)RFP+;(Pax3)GFP+ FMPs and the (MyoD)RFP-;(Pax3)GFP+ FMPs (2×10^4^ cells) were injected into TA muscles of *DMD-null* mice, as described above. Two weeks after engraftment, RFP+ myofibers were observed within the (MyoD)RFP- (Fig. 6B) and (MyoD)RFP+ FMP (Fig. 6C) engrafted TA muscles. Immunostaining showed the expression of dystrophin in RFP+ myofibers derived from both explants (Fig. 6D–I). However, the number of dystrophin+ fibers in TA muscle of the *DMD-null* mice transplanted with MyoD-positive FMPs was higher than that of TA muscle engrafted with MyoD-negative FMPs (50±0.5 fibers and 22.6±1.4 fibers, respectively, *n* = 3 independent experiments; Fig. 6J). These findings demonstrate that engrafted MyoD-positive FMPs had a greater regenerative potential than engrafted MyoD-negative FMPs.

### MyoD-positive FMPs are more Transcriptionally Primed for Myogenesis

**Figure 7:**
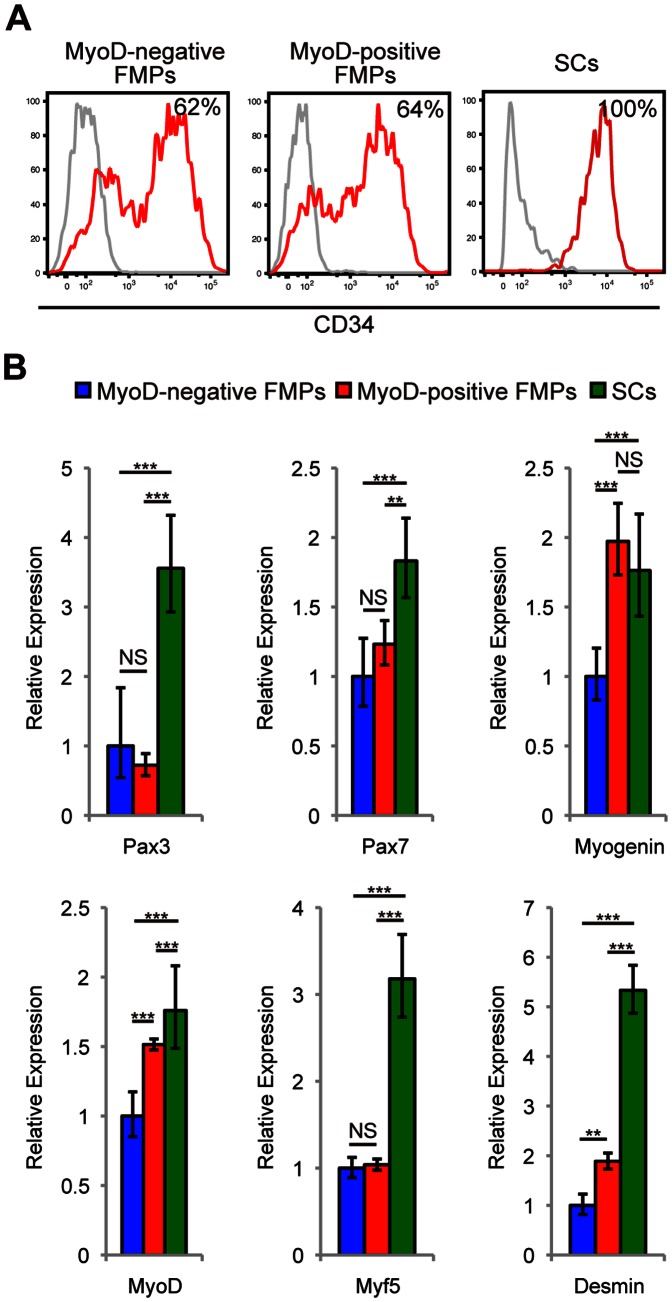
SCs are more primed into the myogenic program than FMPs. (**A**) CD34 expression of (MyoD)RFP- FMPs, (MyoD)RFP+ FMPs, and SCs. Flow cytometry histograms show a control staining profile (gray line) and CD34 (red line). The percentage of cells expressing CD34 is indicated. (**B**) Relative amounts of the indicated transcripts determined by quantitative reverse transcription polymerase chain reaction among (MyoD)RFP- FMPs, (MyoD)RFP+ FMPs, and SCs. Data is normalized to the expression of transcripts for *Rpl13A*. Data is reported as the mean and s.e.m. (*n* = 3). P-values indicated on figures are no significant difference (NS), <0.01 (**), and <0.001 (***). FMPs, fetal skeletal muscle progenitors; SCs, satellite cells.

We hypothesized that the myogenic immaturity of FMPs could affect the efficiency of engraftment, and therefore investigated surface markers, which are known to be expressed in adult SCs. Cxcr4, Sca1, and cMet, which are expressed in some isolated SC populations [30]–[32], were not present in FMPs or (Pax3)GFP+ SCs (Fig. S5). (MyoD)RFP- and (MyoD)RFP+ FMPs are heterogeneous for CD34 expression, which is in marked contrast to its homologous expression in SCs (Fig. 7A).

To evaluate the commitment status of these cells, qRT-PCR was carried out to compare gene expression. *Pax3* transcripts in SCs were higher than that of (MyoD)RFP- and (MyoD)RFP+ FMPs, indicating that GFP expression reflects endogenous Pax3 expression (Fig. 1C,D and Fig. 7B). Transcripts for *Pax7* and *Myf5*, which are reported to be makers of SCs [33], [34], and *Desmin,* a marker for myogenic commitment, were highest in SCs. Transcripts for *MyoD* was higher in SCs and (MyoD)RFP+ FMPs than in (MyoD)RFP- FMPs. Transcripts of *Myogenin*, which is associated with myogenic differentiation, were also higher in (MyoD)RFP+ FMPs and SCs. These transcription profiles imply that SCs are more primed for myogenic commitment than FMPs, and (MyoD)RFP+ FMPs are more primed for the myogenic program than (MyoD)RFP- FMPs.

## Discussion

This study demonstrates the regenerative potential of FMPs, Pax3-expressing skeletal muscle progenitors at the fetal stage. Comparison with (Pax3)GFP+ SCs isolated from adult muscle led us to conclude that FMPs are effective but less efficient in constitution of dystrophin-positive fibers and occupation of the satellite-cell niche in the DMD model mice. Meanwhile, they undergo similar activation of MyoD and muscle differentiation when cultured in vitro (Figs 2, 3), indicating that the myogenic activity of isolated cells in culture is not predictive of their efficiency for regenerative myogenesis after transplantation.

FMPs and SCs have clear differences in their transcriptome profiles. This may partly reflect the fact that adult SCs are quiescent whereas fetal myogenic progenitors are a proliferating population [15]. SCs have higher levels of transcripts for myogenic genes such as *MyoD* and *Myf5* as well as *Pax3* and *Pax7.* Pax7 is required for SC specification while Myf5 is essential for proper regenerative myogenesis [34]–[36]. The analyses of cell surface markers show that 60% of FMPs are positive for CD34 with variable intensity, whereas all SCs express CD34 [14], [27], [33]. Together, the inefficient regenerative ability of FMPs is likely attributed to lower levels of Pax7, Myf5, and CD34 that are involved in myogenic specification, differentiation, and cell adhesion of SCs.

It is well established that SCs are a heterogeneous population [13], especially in terms of Pax3 expression [14], [21]. We used fetal limbs as sources for FMPs, but SCs were collected from abdominal and diaphragm muscles to obtain enough numbers of (Pax3)GFP+ cells for the transplantation because few SCs of limb muscles express Pax3 [14], [21]. We cannot exclude the possibility that the different source of cells between adult and fetal muscles could affect to their transcription profiles and transplantation efficiencies. It would be useful to further investigate whether the SCs from different skeletal muscles might have the different regeneration potentials.

In this study, we also found that part of FMPs in which the MyoD gene had been activated engrafted more efficiently than the rest of FMPs that had not experienced activation of the MyoD gene. MyoD is required for skeletal muscle regeneration in vivo [37]. Genetic tracing experiments, using a *MyoD* Cre with a conditional reporter gene had indicated that most SCs had activated *MyoD* at prenatal stages [29]. Adult SCs indeed continue to transcribe both *Myf5* and *MyoD* when the total (Pax3)GFP+ population is analyzed (Fig. 7B; [38]), which may reflect perduration of GFP in few activated cells that have progressed towards differentiation, although the MyoD protein does not accumulate in the quiescent cells. The MyoD protein starts to accumulate when SCs are activated upon regeneration. In the case of *Myf5*, the low level of Myf5 protein in quiescent SCs has been shown to be due to sequestration of the *Myf5* mRNA, together with miR31 which blocks its activity in mRNP particles [39]. A MicroRNA mediating repression of transcripts required for proliferation has also been reported in quiescent SCs [40]. These observations are consistent with the idea that SCs are primed for rapid proliferation and entry into the myogenic program, which depends on expression of Myf5 and MyoD proteins. When SCs were cultured, they also re-express MyoD protein, but engraft less well than freshly isolated cells [14]. Thus, re-expression of MyoD protein and progression of the myogenic program could affect regeneration potential negatively.

In the FMP population, we also observe transcripts of these myogenic determination genes MyoD and Myf5, although levels are lower than in SCs. In contrast to freshly isolated quiescent SCs, MyoD protein is present in 56% of (Pax3)GFP+ FMPs. This may mainly include population that has entered the myogenic program and will subsequently form new muscle during the extensive growth of the fetal period. A recent report suggests that there are at least two distinct SC populations, one is responsible for the growth of skeletal muscles and another one is activated by muscle injury and survives transplantation [41]. It remains to be elucidated whether FMPs are destined to become one of these two SC populations.

We used a *MyoD-Cre* line that we had developed with a conditional *R26R^RFP^* reporter allele to examine what proportion of FMPs express or had expressed *MyoD* at E16.5. About 50% of the (Pax3)GFP+ cells were RFP+, and most of the RFP+ cells were also MyoD protein-positive. These results indicate that the myogenic progenitor population at this fetal stage has not yet acquired post-transcriptional mechanisms that prevent expression of MyoD protein and that MyoD-negative FMPs do not derive from progenitors that had previously activated this myogenic determination gene. Examination of transcripts in the RFP+ fraction from *MyoD-Cre;R26R^RFP^;Pax3^GFP/+^* of (Pax3)GFP+ showed that the myogenic differentiation gene, *Myogenin*, as well as *Desmin*, an early marker of myogenesis, are also transcribed higher in this population than in the RFP- cells. The RFP+ fraction of the FMPs engrafts better than the RFP- cells that have not yet entered the myogenic program. Our findings therefore underline the importance of myogenic priming in the context of muscle repair and cell therapy for degenerative muscle disease.

Although the therapeutic potentials of SCs are attractive, their regenerative ability is greatly reduced after cell culture, rendering expansion in vitro unsatisfactory [14], [27]. Several studies demonstrated expansion of SCs maintaining engraftment potential [42], [43] but acquisition of enough amounts of SCs for transplantation remains to be a major challenge. Thus other novel cell sources of large quantities of cells are required. Undifferentiated cells like embryonic stem (ES) cells or induced pluripotent stem (iPS) cells are capable of unlimited self-renewal and have the potential to differentiate into any cell type [44]. Myogenic progenitors that form skeletal muscle can be induced from ES or iPS cells in vitro after overexpression of Pax3 or Pax7 [45], [46]. It remains to be elucidated which myogenic populations along the developmental cascade are obtained in such protocols. This is a major issue, highlighted by recent evidence that cardiac progenitors derived from ES cells give rise to immature cardiomyocytes [47], with the risk of arythmias after engraftment into the infarcted heart. Our results show that skeletal muscle progenitors at the fetal stage have the capacity to repair adult skeletal muscle. In addition to demonstrating the regenerative ability of these cells in an adult muscle environment, our findings are of importance to potential ES and iPS derived cell replacement therapies for skeletal muscle.

## Supporting Information

Figure S1 **RFP expression pattern in** ***MyoD-Cre;R26R^RFP^*** **embryos.** (**A**) Schematic representation of MyoD-Cre dependent recombination and derivative reporter alleles. (**B–D**) MyoD-Cre activated expression of RFP from the tdTomato allele and β-galactosidase from *nlacZ* reporter in *R26R^RFP^* mice carrying MyoD-Cre-IRES-nlacZ at E14.5. (**E–G**) RFP-expressing regions co-localize with MyoD labeling in the forelimb at E14.5.
(TIF)

Figure S2 **Immunohistochemistry on serial sections of FMP-engrafted tibialis anterior (TA) muscles, Related to** **Figure 3** **.** (**A–D**) Four serial sections (1500 µm apart between each section, proximal (A) to distal (D)) of a field containing dystrophin+ fibers in the TA muscles of *DMD-null* mice injected with FMPs. Scale bar = 100 µm. FMPs, fetal skeletal muscle progenitors.
(TIF)

Figure S3 **(Pax3)GFP+ cells from embryonic muscle did not show engraftment.** (**A**) *Pax3^GFP/+^* fetus at E10.5 viewed under a fluorescence stereomicroscope. Red dotted lines indicate the dissected region. (**B**) Representative fluorescence-activated cell sorting profiles for (Pax3)GFP+ cells from embryos. (**C**) Forward scatter and side scatter profiles of (Pax3)GFP+ cells gated in (B). (**D,E**) Immunostaining for dystrophin (D) and merged with DAPI (E) in tibialis anterior (TA) muscles of *DMD-null* mice injected with (Pax3)GFP+ isolated from E10.5 embryos 2 weeks after intramuscular engraftment. Scale bars = 100 µm.
(TIF)

Figure S4 **(MyoD)RFP+ cells were positive for MyoD protein, Related to** **Figure 6** **.** (**A**) Gating strategy to isolate (MyoD)RFP- and (MyoD)RFP+ FMPs. GFP and RFP expressing cells from wild-type, *Pax3^GFP/+^*, *MyoD-Cre;R26R^RFP^*, and *Pax3^GFP/+^;MyoD-Cre;R26R^RFP^* mice. (**B–I**) Immunocytochemistry of isolated (MyoD)RFP- and (MyoD)RFP+ FMPs for RFP (B,F), GFP (C,G), MyoD (D,H), and DAPI (E,I). Scale bar = 50 µm. FMPs, fetal skeletal muscle progenitors.
(TIF)

Figure S5 **Surface marker profiles of FMPs and SCs, Related to** **Figure 7** **.** FMPs and SCs were negative for Cxcr4, Sca1, and cMet. FMPs, fetal skeletal muscle progenitors; SCs, satellite cells.
(TIF)

Table S1 **Primers used for the expression analysis of the indicated gene by qPCR, Related to Materials and Methods.**
(DOC)
