# Supplementary figures and images for: Fetal Skeletal Muscle Progenitors Have Regenerative Capacity after Intramuscular Engraftment in Dystrophin Deficient Mice
Source: PLoS One. 2013 May 9;8(5):e63016. doi: 10.1371/journal.pone.0063016 (PMC3650009; doi:10.1371/journal.pone.0063016)

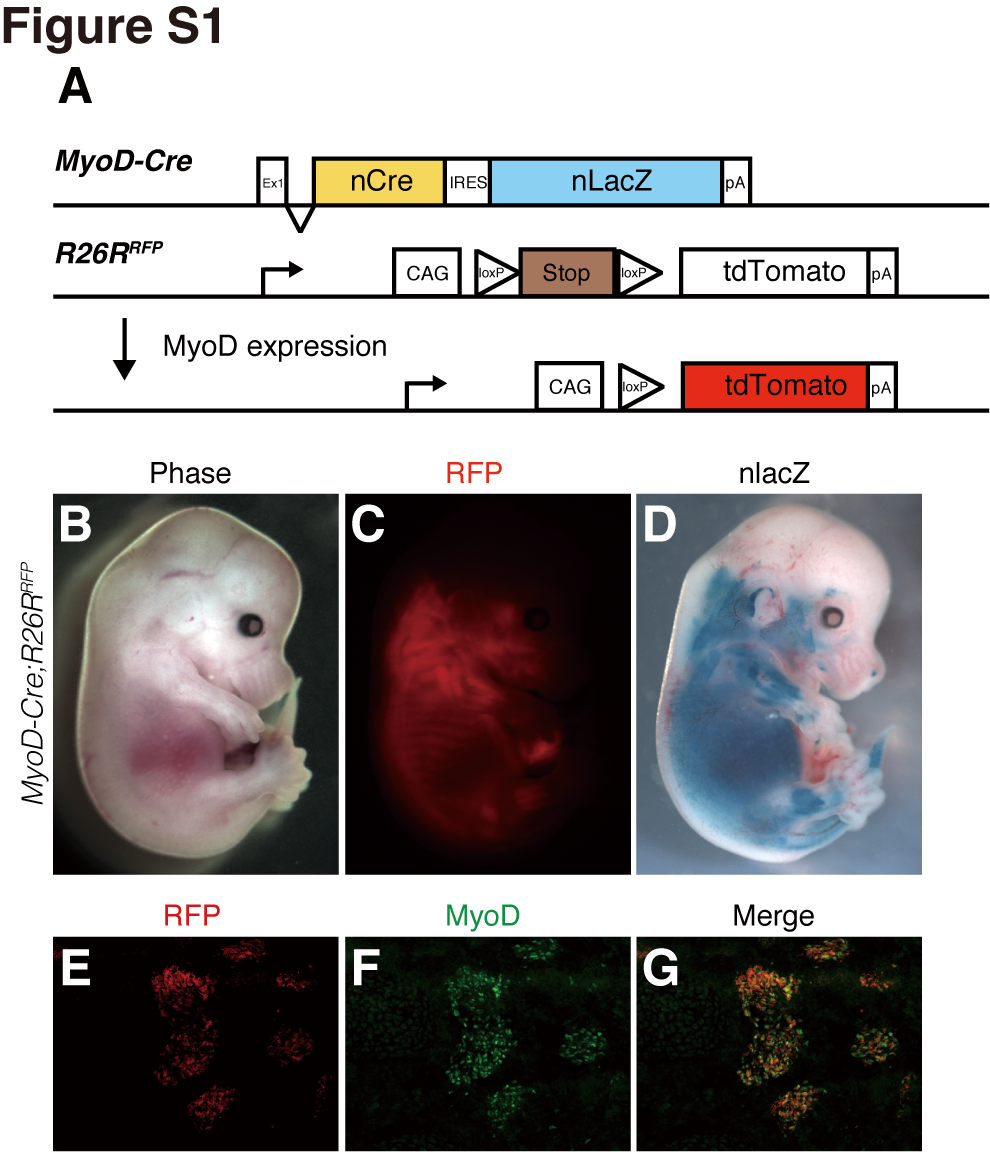

Supplement: Figure S1 — RFP expression pattern in MyoD-Cre;R26RRFP embryos. (A) Schematic representation of MyoD-Cre dependent recombination and derivative reporter alleles. (B–D) MyoD-Cre activated expression of RFP from the tdTomato allele and β-galactosidase from nlacZ reporter in R26RRFP mice carrying MyoD-Cre-IRES-nlacZ at E14.5. (E–G) RFP-expressing regions co-localize with MyoD labeling in the forelimb at E14.5. (TIF) [file pone.0063016.s001.tif]

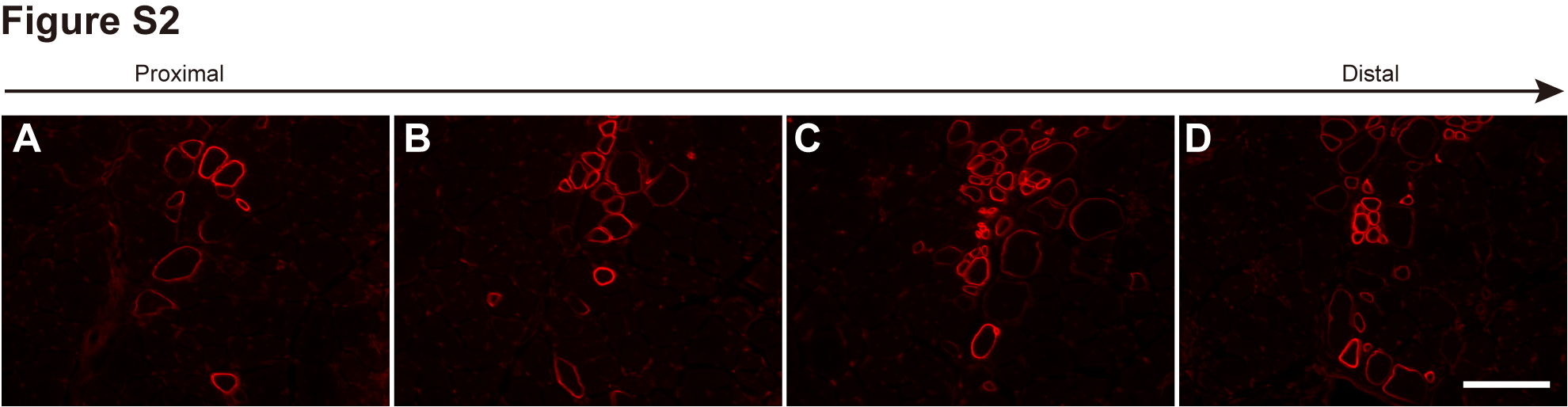

Supplement: Figure S2 — Immunohistochemistry on serial sections of FMP-engrafted tibialis anterior (TA) muscles, Related to Figure 3 . (A–D) Four serial sections (1500 µm apart between each section, proximal (A) to distal (D)) of a field containing dystrophin+ fibers in the TA muscles of DMD-null mice injected with FMPs. Scale bar = 100 µm. FMPs, fetal skeletal muscle progenitors. (TIF) [file pone.0063016.s002.tif]

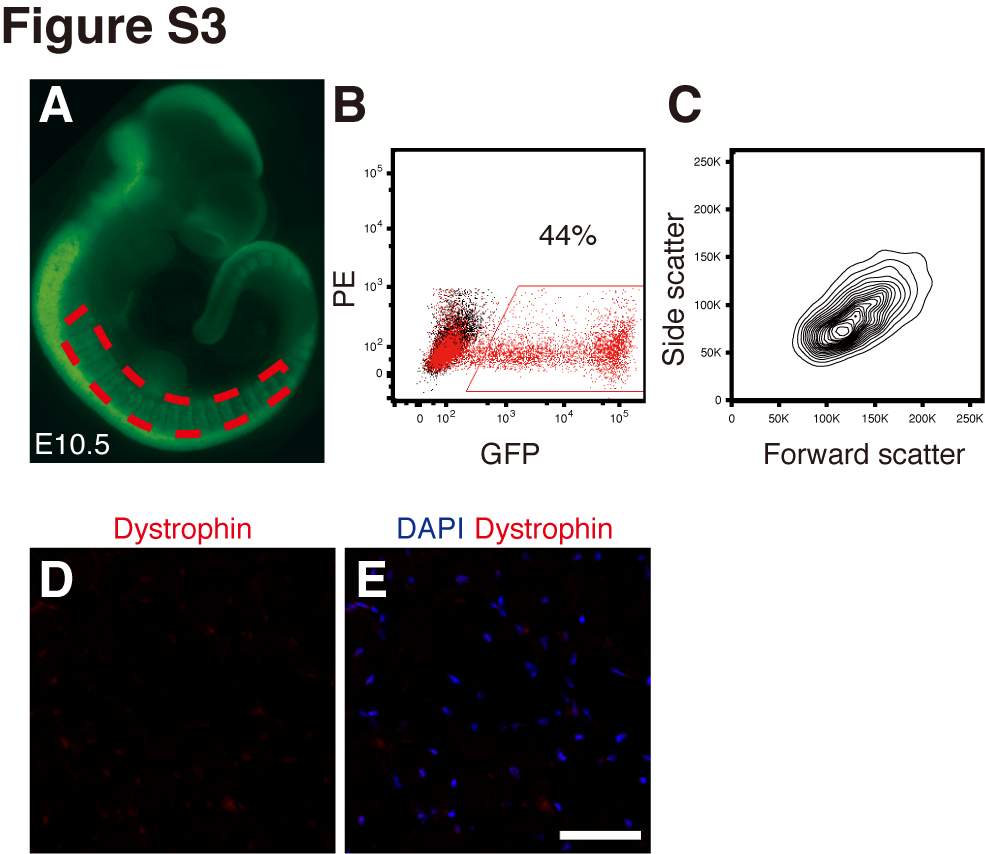

Supplement: Figure S3 — (Pax3)GFP+ cells from embryonic muscle did not show engraftment. (A) Pax3GFP/+ fetus at E10.5 viewed under a fluorescence stereomicroscope. Red dotted lines indicate the dissected region. (B) Representative fluorescence-activated cell sorting profiles for (Pax3)GFP+ cells from embryos. (C) Forward scatter and side scatter profiles of (Pax3)GFP+ cells gated in (B). (D,E) Immunostaining for dystrophin (D) and merged with DAPI (E) in tibialis anterior (TA) muscles of DMD-null mice injected with (Pax3)GFP+ isolated from E10.5 embryos 2 weeks after intramuscular engraftment. Scale bars = 100 µm. (TIF) [file pone.0063016.s003.tif]

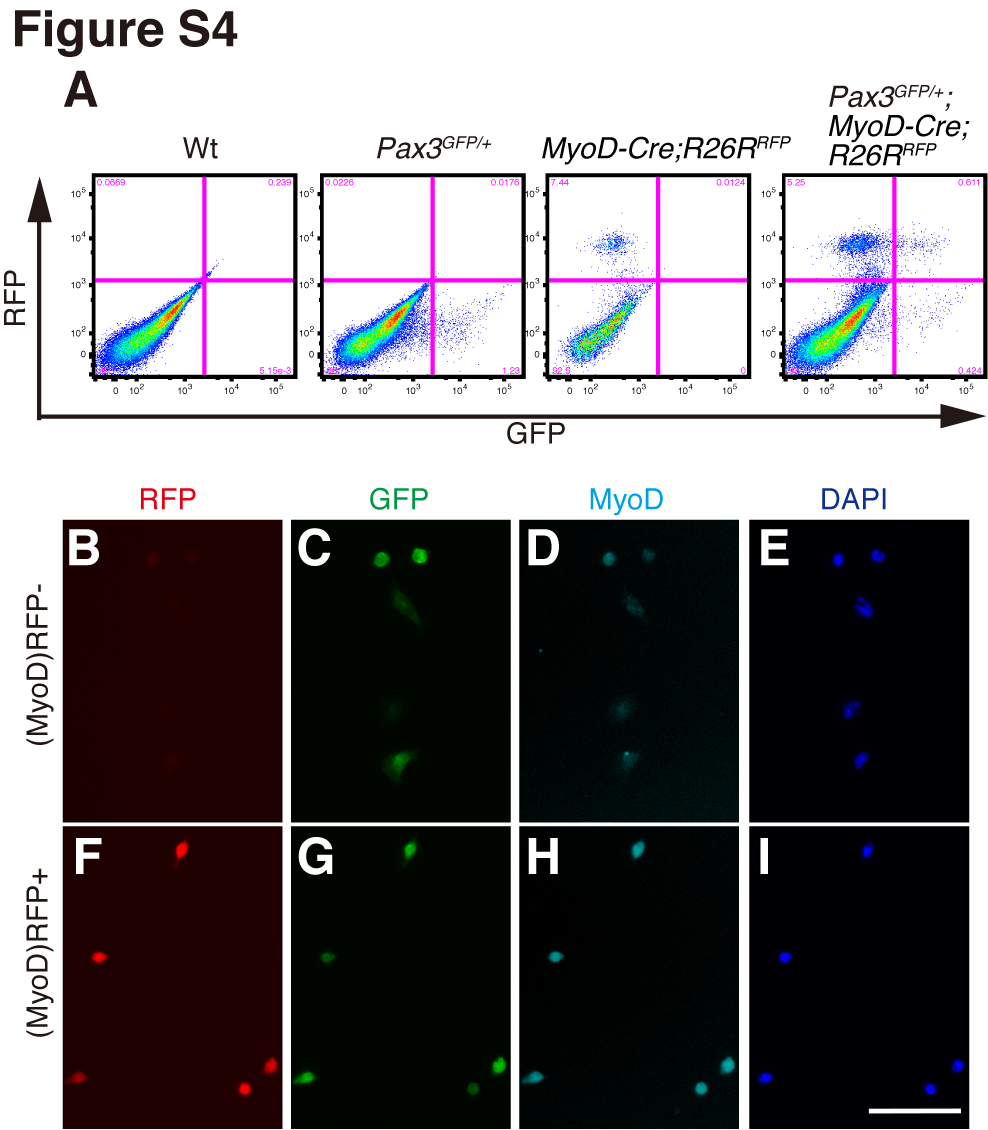

Supplement: Figure S4 — (MyoD)RFP+ cells were positive for MyoD protein, Related to Figure 6 . (A) Gating strategy to isolate (MyoD)RFP- and (MyoD)RFP+ FMPs. GFP and RFP expressing cells from wild-type, Pax3GFP/+, MyoD-Cre;R26RRFP, and Pax3GFP/+;MyoD-Cre;R26RRFP mice. (B–I) Immunocytochemistry of isolated (MyoD)RFP- and (MyoD)RFP+ FMPs for RFP (B,F), GFP (C,G), MyoD (D,H), and DAPI (E,I). Scale bar = 50 µm. FMPs, fetal skeletal muscle progenitors. (TIF) [file pone.0063016.s004.tif]

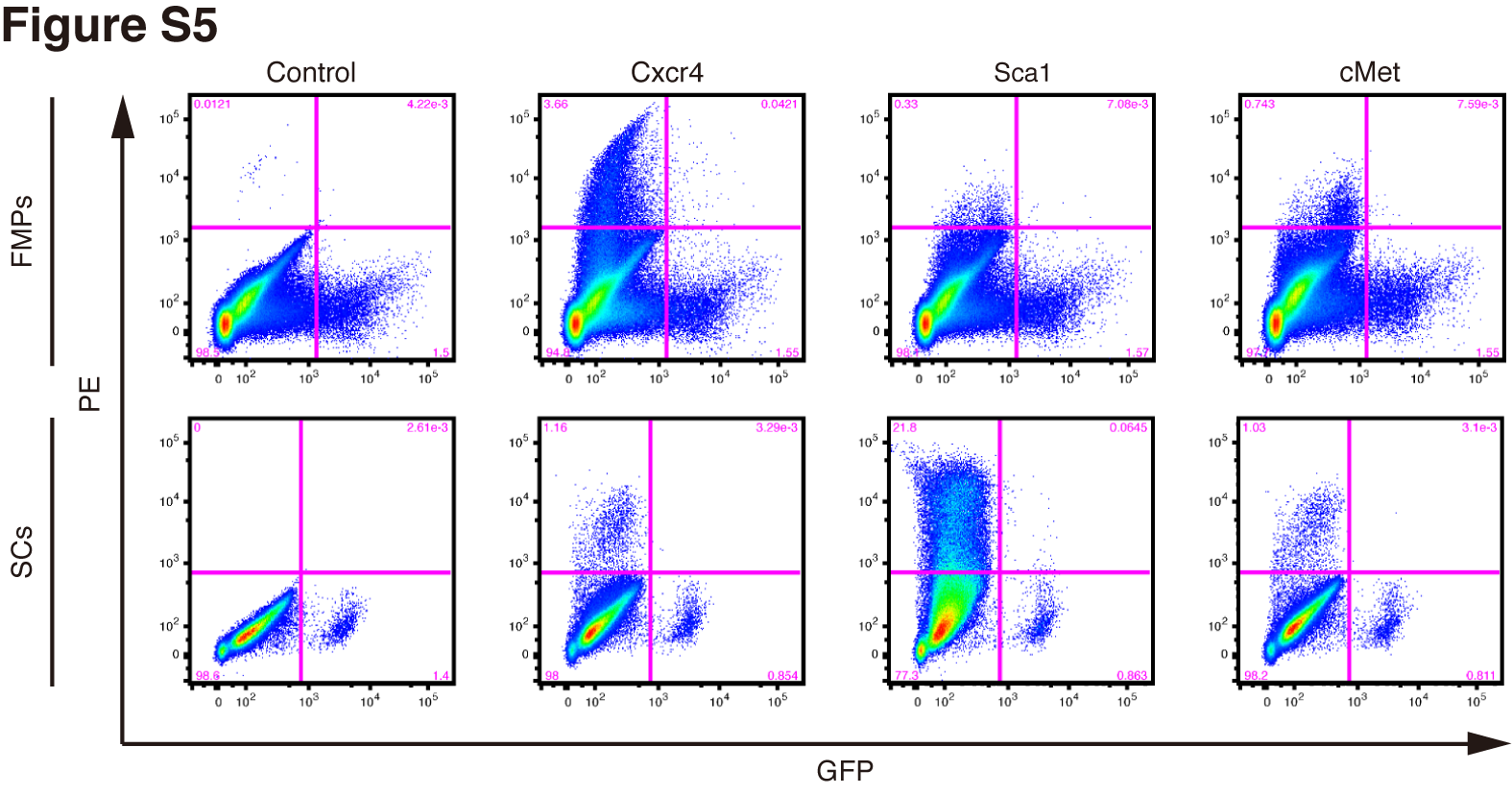

Supplement: Figure S5 — Surface marker profiles of FMPs and SCs, Related to Figure 7 . FMPs and SCs were negative for Cxcr4, Sca1, and cMet. FMPs, fetal skeletal muscle progenitors; SCs, satellite cells. (TIF) [file pone.0063016.s005.tif]
