# Supplementary material for: Fetal Skeletal Muscle Progenitors Have Regenerative Capacity after Intramuscular Engraftment in Dystrophin Deficient Mice
Source: PLoS One. 2013 May 9;8(5):e63016. doi: 10.1371/journal.pone.0063016 (PMC3650009; doi:10.1371/journal.pone.0063016)
Supplement: Table S1 — Primers used for the expression analysis of the indicated gene by qPCR, Related to Materials and Methods. (DOC) [file pone.0063016.s006.doc]

**Table S1.** Primers used for the expression analysis of the indicated gene by qPCR.

| **Target Gene** | **Forward primer** | **Reverse primer** |
| --- | --- | --- |
| *Desmin* | TACACCTGCGAGATTGATGC | ACATCCAAGGCCATCTTCAC |
| *Myf5* | CCACCTCCAACTGCTCTGA | GCTGTCAAAGCTGCTGTTCTT |
| *Myod1* | AGCACTACAGTGGCGACTCA | GGCCGCTGTAATCCATCAT |
| *Myog* | CAACCAGGAGGAGCGCGATCTCCG | GGCGCTGTGGGAGTTGCATTCACT |
| *Pax3* | TCCATCCGACCTGGTGCCAT | TTCTCCACGTCAGGCGTTG |
| *Pax7* | AGGCCTTCGAGAGGACCCAC | CTGAACCAGACCTGGACGCG |
| *Rpl13A* | GTGGTCCCTGCTGCTCTCAAG | CGATAGTGCATCTTGGCCTTTT |
